# Spatial heterogeneity in mass drug administration from a longitudinal epidemiological study assessing transmission interruption of soil transmitted helminths in the Wolaita zone of southern Ethiopia (Geshiyaro Project)

**DOI:** 10.1371/journal.pntd.0011947

**Published:** 2024-02-08

**Authors:** Santiago Rayment Gomez, Rosie Maddren, Ewnetu Firdawek Liyew, Melkie Chernet, Ufaysa Anjulo, Adugna Tamiru, Getachew Tollera, Geremew Tasew, Birhan Mengistu, Benjamin Collyer, Kathryn Forbes, Roy Anderson

**Affiliations:** 1 London Centre for Neglected Tropical Disease Research, Department of Infectious Disease Epidemiology, Faculty of Medicine, St Marys Campus, Imperial College London, London, United Kingdom; 2 Bacterial, Parasitic and Zoonotic Disease Research Directorate, Ethiopian Public Health Institute, Addis Ababa, Ethiopia; 3 Disease Prevention and Health Promotion Core Process, Ministry of Health, Wolaita, Ethiopia; George Washington University School of Medicine and Health Sciences, UNITED STATES

## Abstract

**Objectives:**

Deworming programmes of soil-transmitted helminths are generally monitored and evaluated by aggregating drug coverage and infection levels at a district level. However, heterogeneity in drug coverage at finer spatial scales means indicators may remain above thresholds for elimination as a public health problem or of transmission in some areas. This paper aims to highlight the misleading information that aggregating data at larger spatial scales can have for programme decision making.

**Methods:**

Drug coverage data from the Geshiyaro project were compared at two spatial scales with reference to the World Health Organisation’s targets. District (woreda) and village (kebele) level were compared. The association between infection levels and drug coverage was analysed by fitting a weighted least-squares function to the mean intensity of infection (eggs per gram of faeces) against drug coverage.

**Results:**

The data show clearly that when the evaluation of coverage is aggregated to the district level, information on heterogeneity at a finer spatial scale is lost. Infection intensity decreases significantly (p = 0.0023) with increasing drug coverage.

**Conclusion:**

Aggregating data at large spatial scales can result in prematurely ceasing deworming, prompting rapid infection bounce-back. There is a strong need to define context-specific spatial scales for monitoring and evaluating intervention programmes.

## Author summary

For soil-transmitted helminths (STH), control is centred around mass drug administration (MDA) of eligible subgroups without prior diagnosis, aiming to achieve 75% drug coverage. MDA programmes are monitored by assessing whether drug coverage and infection prevalence and intensity, aggregated to a spatial scale, typically an administrative unit such as a district, have met programmatic targets. Spatial heterogeneity in drug coverage, prevalence, and intensity is commonplace and well documented. However, little attention has been paid to the spatial scales of this heterogeneity and the implications it has on providing the basis to inform the cessation or continuation of prophylactic mass treatment. We present a clear analysis showing that fine scale spatial heterogeneity in drug coverage is commonplace even in well implemented MDA programmes. We show that aggregating data at the district level, which is routine for deworming programmes, can give misleading information on which to base future treatment decisions, specifically the premature reduction in frequency, or cessation of MDA, because fine scale heterogeneity is not captured by larger aggregations. This work highlights the critical need to define context specific spatial scales for monitoring and evaluating interventions implemented in different transmission settings.

## Introduction

Soil-transmitted helminths (STH) are a group of intestinal parasites whose mode of transmission is primarily through direct contact, or ingestion, of soil contaminated with infective eggs or larvae [1]. They are geographically distributed in tropical and subtropical regions with focal points of transmission in the most socio-economically deprived populations. Specifically, these most disadvantaged populations are predominantly burdened by the estimated 1.5 billion global infections [2] STHs are targeted for elimination as a public health problem by the World Health Organisation (WHO), defined as achieving less than 2% of moderate or heavy intensity infections. To reach this target, periodic mass drug administration of albendazole or mebendazole to pre-school-aged children (pre-SAC), school-aged children (SAC), women of reproductive age (WRA) and those in what are considered to be high-risk occupations (e.g., agriculturalists for hookworm), is recommended with a coverage target of 75% of the eligible population swallowing the treatment [3,4]

In contexts where MDA is being implemented to eliminate STH as a public health problem, performance indicators (e.g., drug coverage levels) and impact indicators (e.g., prevalence and intensity of infection), are aggregated to a defined spatial scale, which is typically an administrative unit such as a district, as a means of monitoring the intervention [5]. After periodic MDA with good coverage, the prevalence and intensity of infection indicators collected from sentinel sites, aggregated at the district level, are likely to decrease and may indicate that overall prevalence has decreased sufficient enough to reduce the frequency of MDA rounds. However, it is well recognised that spatial heterogeneity in prevalence and intensity of STH infection is commonplace and as such, hotspots of infection can be important drivers of transmission even when average levels are low [6] Thus, it is highly probable that existing heterogeneity means that in particular areas, at finer spatial scales, the prevalence and intensity are likely to remain above this threshold and the premature altering of MDA strategies may induce a rapid infection bounce-back to pre-intervention levels. Variation in MDA coverage is itself an important driver of observed spatial heterogeneity in prevalence and intensity of STH infection [7] While substantial coverage heterogeneity has been documented [8–10]]. factors driving this variability are not well understood and very little has been discussed about the consequences for control and elimination of using large spatial scales for monitoring and evaluation (M&E). Furthermore, current estimates for the coverage and frequency of MDA are derived from non-spatially structured transmission models which do not consider heterogeneity in coverage between connected geographical areas [6].

Here, we use MDA coverage data collected in year 4 of the Geshiyaro project in Southern Ethiopia and its coverage evaluation surveys (CES) to highlight the importance of needing to define an adequate, context-specific, and possibly dynamic, spatial scale across which to evaluate the progress of interventions aimed at eliminating STH as a public health problem. Factors involved with choosing an appropriate spatial scale are discussed. Detailed descriptions of the overall design and aims of the Geshiyaro project are recorded in previous publications [11]

## Methods

### Ethics statement

Data were collected as part of the Geshiyaro project, a longitudinal STH elimination feasibility study, the protocol of which has been previously published [11]. The study received ethical approval from the Institutional Review Board (IRB) at the Scientific and Ethical review Office of the Ethiopian Public Health Institute. Formal consent was obtained verbally from all individuals and for children, a parent provided formal verbal consent.

### Study site and design

The Geshiyaro project is set in the Wolaita zone of south-western Ethiopia, where there are an estimated two million inhabitants living across 23 districts (woreda). The project has three intervention arms designed to assess which combination(s) of interventions are most effective at reducing STH prevalence. Arm one interventions involve community-wide MDA, improved WaSH infrastructure, and behavioural change communication (BCC), arm two involves community-wide MDA and the existing national ‘one WaSH’ programme and arm three involves school-based MDA and the existing national ‘one WaSH’ programme. Detail can be found in the protocol paper previously published [11]. A census was carried out in a sample of five districts (three from arm one and two from arm two) prior to any interventions. All household participants were registered using biometric fingerprint scanning and/or subject ID cards. During subsequent project activities, such as MDA, participants were linked longitudinally by accessing their 11-digit identification number either through biometric fingerprint scanning or from the physical ID card. Individuals not enrolled during the census were newly registered and added to the population census database during these project activities. The subsequent analyses use data collected from the five districts where biometric registration was employed and monitors over 600,000 individuals. Enrolment status did not prevent anyone from accessing deworming medication.

### Mass drug administration

Albendazole was intended to be administered annually to all districts of the Wolaita zone, however the delivery method was increased to biannual, to account for delayed MDA rounds during the height of the Covid-19 pandemic. Distribution was also changed from fixed-point to house-to-house to avoid mass gatherings. During each MDA round, all consenting individuals approached by a health extension worker (HEW) were identified by their fingerprint (biometric), subject ID, or name previously recorded during census activities. All individuals were offered treatment regardless of their infection status and individuals who did not consent to having a biometric taken were also offered treatment as usual as part of the national deworming programme. For participants who accepted treatment, the aim was for HEWs to record whether treatment was directly observed. The definition of MDA coverage used herein is the percentage of eligible participants who accepted albendazole.

### Spatial data preparation

The delineation of administrative units in Ethiopia is highly variable, particularly in the Southern Nations, Nationalities and Peoples region. Redistricting at all administrative unit levels is commonplace, but the latest changes are not regularly updated and thus reflected in available geo-referenced data. Additionally, there are often discrepancies in the spellings of administrative unit names attributed to the difficulty in translating Amharic and local languages to Latin scripture. Data management alongside current Ministry of Health (MoH) information allowed existing shapefile data (available from https://gadm.org/download_country.html) for the Wolaita zone to be manipulated in such a way that MDA coverage for all current villages (or cluster of villages) could be mapped. In some instances, what was previously a single village cluster (and represented by a single spatial polygon in shapefile data) has now been split into multiple clusters. In such cases, MDA coverage for the villages that make up a single village was aggregated to allow for mapping. In other instances, villages have become town administrations (distinct administrative units in Ethiopia) or been incorporated into new districts, which do not fall under the reach of the Geshiyaro Project. In these instances, data from the coverage evaluation surveys (CES)–designed to provide robust drug coverage estimates and overcome limitations of reported coverage–conducted by Ethiopian Public Health Institute (EPHI) staff following a round of MDA implemented by the Geshiyaro project, were used to obtain complete maps [12]. As a result, CES coverage estimates were generated from a different denominator, yet still representative of the Geshiyaro census denominator. Results clearly indicate the few instances where validated coverage data could not be identified for an administrative unit.

### Statistical analyses

All analyses and figures were computed in RStudio (R version 4.1.2). Shapefile data were obtained from the Database of Global Administrative Areas. Participants were grouped into age brackets: Pre-SAC (2 – 4yrs), SAC (5 – 14yrs) and adolescents and adults (>14yrs). For the coverage maps, percentage standard errors were calculated and 95% confidence intervals using the Clopper-Pearson method were calculated for arithmetic mean prevalence. For the association between infection intensity (eggs per gram of faeces [EPG]) and coverage, bootstrapped sampling (r = 1000) with replacement was employed for calculating mean intensity and 95% confidence intervals [13].

## Results

**Fig 1:**
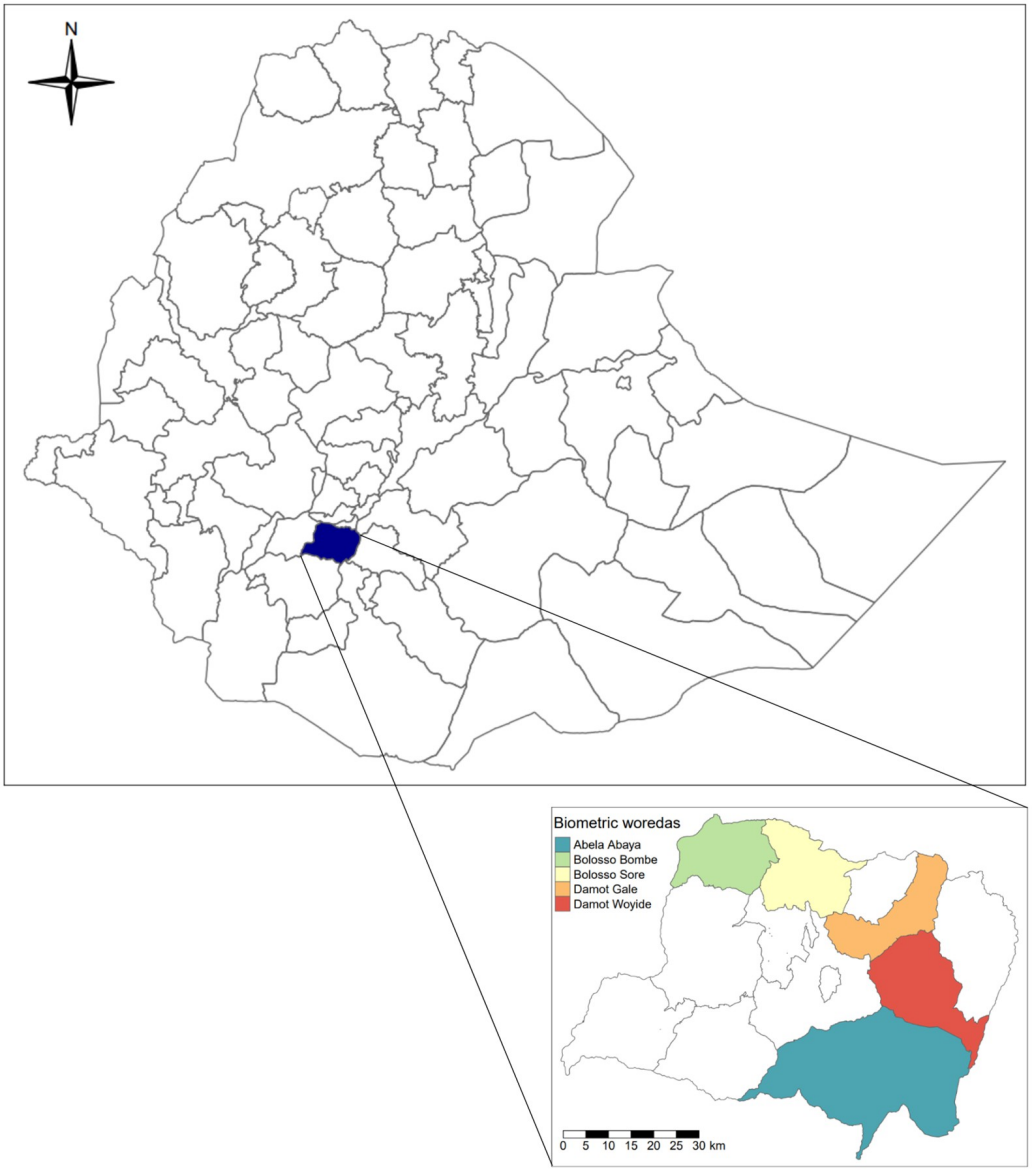
Map of Ethiopia showing the location of the Geshiyaro study region in southwestern Ethiopia. Coloured polygons in the inset map highlight the five districts that were censused and biometrically monitored. Shapefile data from GADM (https://gadm.org/download_country.html).

Biometrically verified MDA coverage data was available for 92 villages and coverage evaluation survey (CES) survey data was used for the remaining 35 villages that were not under the remit of Geshiyaro MDA. The villages fall within the five censused districts (Fig 1).

**Fig 2:**
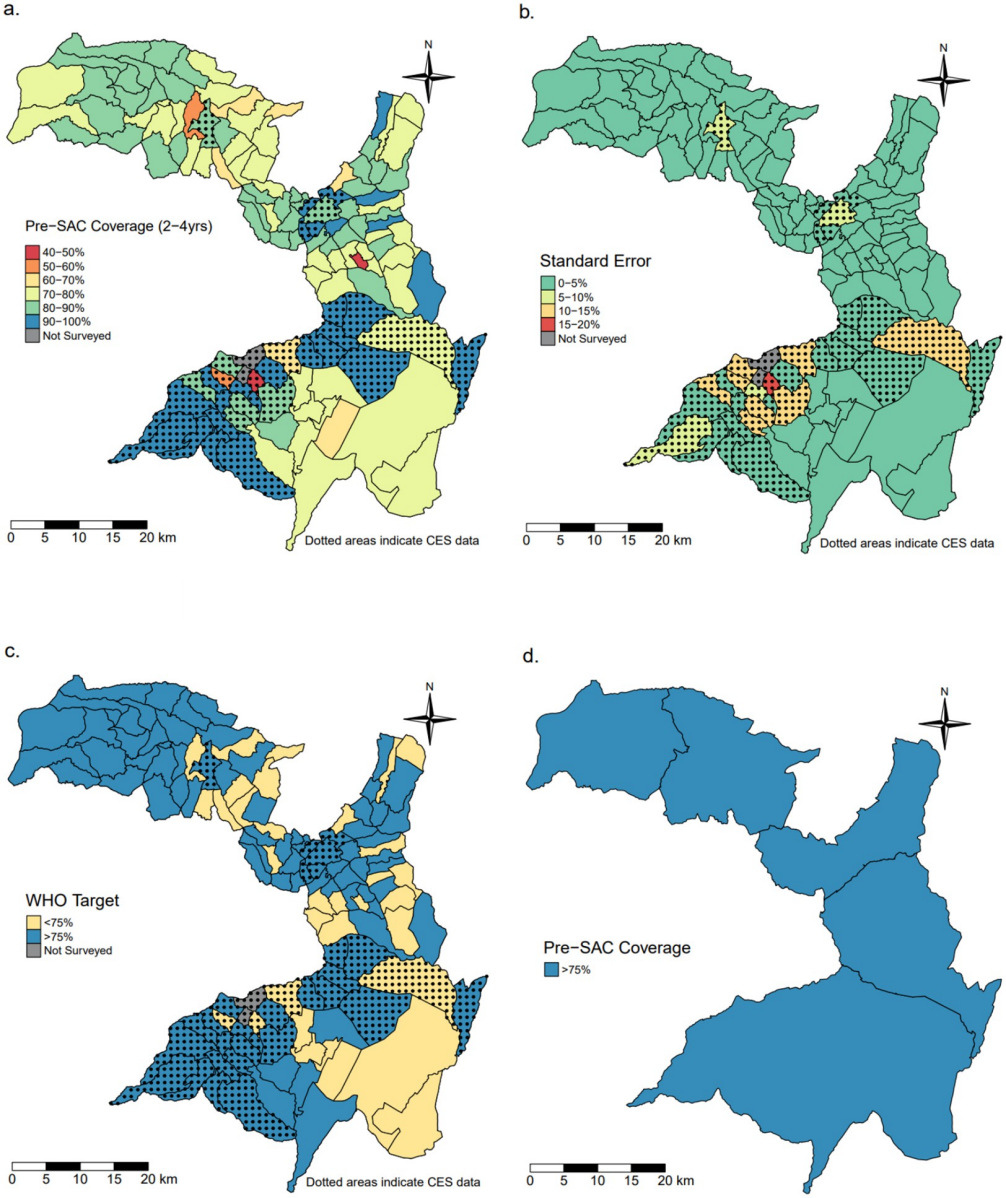
Maps of the Geshiyaro study region within the Wolaita zone. (a) village level MDA coverage in pre-School Aged Children (Pre-SAC) populations; (b) standard error of MDA coverage; (c) villages that were above or below WHO’s target threshold for MDA coverage (75%); (d) the aggregated MDA coverage at the district level. Abbreviations: Pre-SAC–pre-school-aged children. Shapefile data from GADM (https://gadm.org/download_country.html).

There was substantial heterogeneity in Pre-SAC coverage with a range from 40% to 100% and a mean of 82∙2% (SE = 2∙73) across the entire area at the village level. Overall, a quarter of the villages (33/127) did not meet the 75% coverage targets set out by the WHO for elimination as a public health problem. However, when aggregated to the district level, coverage was >75% for all woredas, with a range of 77∙3% to 84∙7% and mean of 81∙9% (SE = 0∙32) (Fig 2).

**Fig 3:**
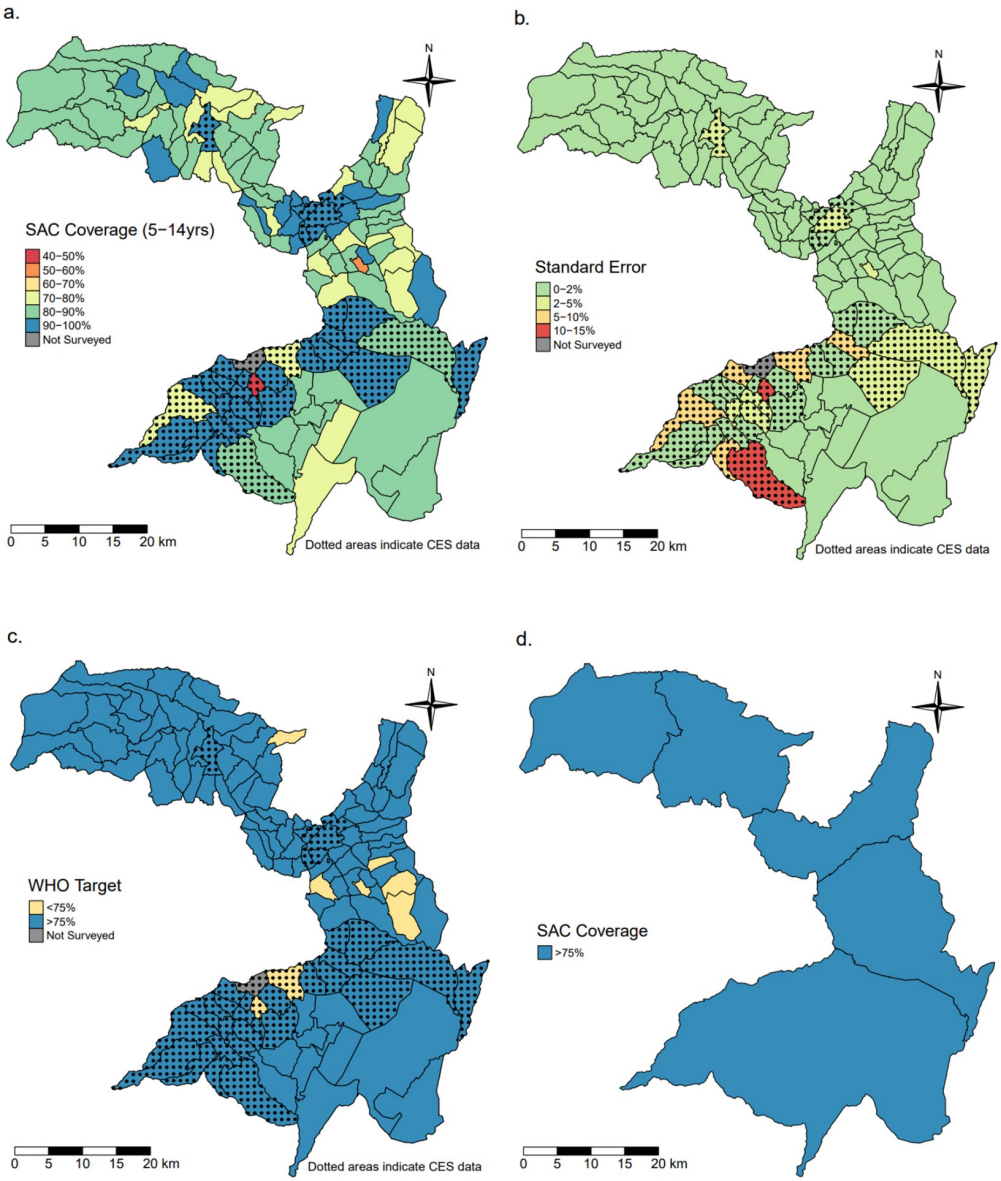
Maps of the Geshiyaro study region within the Wolaita zone. (a) village level MDA coverage in School Aged Children (SAC) populations; (b) standard error of MDA coverage; (c) villages that were above or below WHO’s target threshold (75%) for MDA coverage; (d) the aggregated MDA coverage at the district level. Abbreviations: SAC–school-aged children. Shapefile data from GADM (https://gadm.org/download_country.html).

In SAC, heterogeneity in coverage also existed at the village level, ranging between 45∙4% and 100% with a mean of 87∙0% (SE = 1∙47). In SAC, just over 5% (8/127) of villages did not meet the 75% coverage targets and coverage was >75% in all districts when aggregated at the district level, with a range of 83∙6% to 89∙6% and a mean of 86∙5% (SE = 0∙18) (Fig 3).

**Fig 4:**
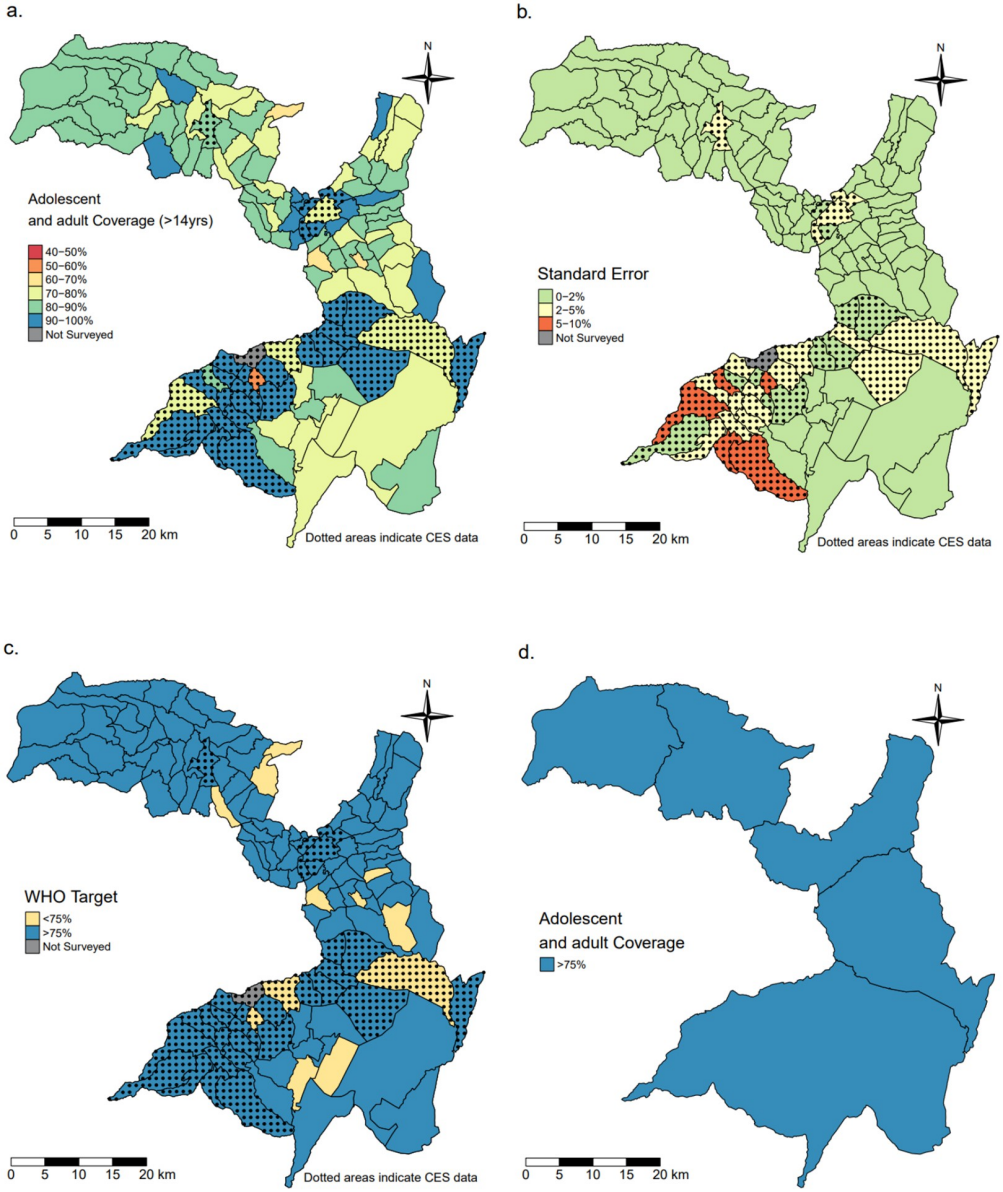
Maps of the Geshiyaro study region within the Wolaita zone. (a) village level MDA coverage in adult populations; (b) standard error of MDA coverage; (c) villages that were above or below WHO’s target threshold for MDA coverage; (d) the aggregated MDA coverage at the district level. Shapefile data from GADM (https://gadm.org/download_country.html).

In adolescents and adults (>14 years), heterogeneity at the village level existed within a narrower range of 58∙8% to 85∙1% and with a mean of 83.9% (SE = 1∙37). Approximately a tenth of villages (12/127) did not meet the 75% coverage targets but surpassed the 75% target when aggregated at the district level, with a range of 82∙2% to 87∙7% and a mean of 84∙7% (SE = 0∙44) (Fig 4).

On average, CES coverage (90∙5%; 95% CI: 76∙5%– 96∙7% tended to be higher than the Geshiyaro’s biometrically verified coverage (82∙1%; 95% CI: 79∙8%– 84∙4%), however, the overlapping 95% confidence intervals suggest there was no statistically significant difference between the means. Similarly, comparing the district and age stratified coverage estimates for the two data sources showed overlapping 95% confidence intervals (S1 Fig). As such, this difference was not considered important for further investigation.

## Discussion

Assessing the success of deworming programmes is dependent on an ability to accurately measure key indicators, such as MDA coverage and infection prevalence and intensity. The WHO has set out an MDA coverage target of 75% in pre-SAC, SAC and WRA with the aim of eliminating STH as a public health problem. However, no guidance is given for fine spatial scale reporting of these metrics.

The Geshiyaro project data on MDA coverage shows clearly that when the evaluation of coverage is aggregated up to the district level, information on heterogeneity at a finer spatial scale (i.e., village level) is lost, leading to poorly informed M&E and downstream programmatic efficacy. Panel D in Figs 2–4 illustrate that while coverage exceeds the 75% WHO target in all districts for all age groups, there are obvious pockets of low coverage (40–60%) in the finer spatial scale of villages (Panel A). After four years of MDA, *A*. *lumbricoides* infection intensities were all classed as low intensity according to WHO guidance (1–4,999 EPG) [4]. While significant reductions in infection intensities may be made with increasing coverage at the district level, some relatively higher infection intensities likely remain,. seeded by villages within a district with lower coverage and, concomitantly, higher infection prevalence and intensity. From a public health policy perspective, the implication of this is that failing to monitor intervention progress at finer spatial scales means national control programs could prematurely reduce or stop MDA due to the false conclusion that coverage targets aggregated at less granular spatial scales have been uniformly met across small spatial scale units such as a village(s). For example, the Expanded Special Project for Elimination of Neglected Tropical Diseases (ESPEN), which was established by the WHO Regional Office for Africa to accelerate elimination of the five most prevalent NTDs, summarises data at the district level which may inadvertently suggest more progress has been made towards these goals than is strictly the case if fine scale pockets of high infection persist. Small pockets of medium or high infection prevalence and intensity in a landscape of low average infection levels can potentially sustain transmission across much larger spatial scales. The constraints of aggregating data are suitably shown by Truscott *et al* [14,15] in data analysis for the DW3 project on STH control in three countries [16] who found that while the mean prevalence of a cluster of communities may be below a given threshold, some individual communities may be above it and this decreased the likelihood of interrupting transmission. In fact, based on parameter estimates from the DW3 study, the stochastic individual based simulations showed that a collection of communities in a cluster failing to reach the elimination threshold, and with prevalence above the cluster mean, will likely bounce back to endemicity, resulting in the whole cluster failing to interrupt transmission as predicted [14]

Ideally, M&E would be carried out at the smallest administrative unit, however, this is resource intensive so to a large extent, funding availability dictates how fine a scale M&E can be carried out at. Nonetheless, a future strategy that involves identifying hotspots of high prevalence driven by high rates of reinfection [17]. and targeting MDA to these areas means performance and impact indicators can be monitored at the finest scale possible, removing the need for a blanket, resource intensive M&E strategy. Hotspots of high prevalence are important for sustaining transmission [15,18,19] and thus if they could be prioritised for MDA, the opportunities for elimination, whether as a public health problem or transmission interruption, are increased. This practice has already begun to be implemented for schistosomiasis [20,21]. Recently a framework of diagnostic use-cases and target product profiles (TPP) was developed to categorise transitions between control programme phases [22,23]. A similar framework should be developed that defines adequate spatial scales for M&E as programmes move from morbidity control to elimination as transmission interruption, using hotspots that need treatment targeting to define the *use-case* for programmes in low transmission settings.

As countries shift their focus to the possibility of eliminating transmission, a critical question that remains unanswered is what is an appropriate spatial unit to define STH transmission within and between adjacent units? For a clearly defined spatial unit with a known pristine transmission intensity level prior to widescale MDA, a formal coverage target should exist for the elimination or cessation of parasite transmission. At present none exists. Based on mathematical models of parasite transmission and MDA impact with parameters derived from data [15] the Geshiyaro project, which aims to assess the feasibility of transmission interruption, has set a drug coverage target of 90% across all age groups to achieve this goal. Nevertheless, Anderson and others [14,15,19] note the ambiguities surrounding these proposed threshold targets. For example, transmission interruption depends on the initial baseline prevalence and average intensity of infection (both determined by the magnitude of the basic reproductive number R_0_). Furthermore, regardless of the initial transmission intensity, achieving transmission interruption with prediction models of parasite transmission and MDA impact occurs only in the absence of movement of infected individuals from neighbouring villages or districts who can shed infective stages into the environment [14,19]. In reality this assumption does not hold true. In a given setting, the spatial unit over which transmission occurs depends on many factors including urbanisation, transportation routes, location of schools and health care facilities, and human mobility. The likelihood that parasites will be reintroduced following successful transmission interruption in a defined location will depend on the linkages of the area with other locations which may or may not have reached the conditions required to stop transmission. The influence of these factors will vary greatly in different geographical and cultural settings. Our understanding of these factors and variation of them in different contexts is very limited at present. This may help explain why WHO policy on defining what spatial scales MDA coverage levels should be reported is so vague at present. What can be done to improve knowledge in this area?

Whilst non-spatially structured transmission models can propose probabilities of elimination or bounce-back under various scenarios [15,24] the reality of identifying transmission breakpoints in different settings must include the coupling of spatial movement patterns with parasite genetic data and molecular epidemiological analyses. Identifying variable genome regions creates the opportunity to ascertain ‘who infects whom’ over different spatial scales, for example, within households, within and between households in a village, and between villages Add citation. Sampling the genetic material of expelled adult STH worms post-MDA via worm expulsion studies and associated phylogenetic analyses, offers the ability to describe the STH genomic landscape in a geographical region [25,26]

Recent stochastic mathematical modelling studies of STH transmission and control within a spatially heterogeneous environment in Kenya [27] show that the correct spatial scale at which to evaluate intervention progress does indeed depend on the movement patterns of people as linked to the spatial dispersal of infective stages in defined social settings, as does the likelihood and degree to which transmission will persist. Our understanding of how STH transmission, as well other NTDs, is affected by spatial scale is very poor at present but molecular epidemiological studies would act to remedy this situation.

On average, the villages achieving the highest coverage tended to be where CES data was used instead of Geshiyaro data (Figs 2–4). Without an in-depth analysis, it is unclear whether the CES methodology [4] generated an overestimation of coverage. The general concern for survey-based data collection as suggested by WHO for the CES surveys, is treatment recall bias from participants, and health workers exaggerating coverage which can both lead to overestimation [28,29].

One limitation of this study arises from the two different data sources used to generate coverage maps. On the one hand, MDA coverage from Geshiyaro data is biometrically verified and based on directly observed treatment, whilst CES coverage is based on participants self-reporting drug swallowing, which is known to be subject to substantial bias. Moreover, the denominators used for the coverage calculations of each data source differ; Geshiyaro denominators represent the entire eligible population for an administrative unit, whilst CES denominators represent the total number of participants surveyed, i.e., a sub-sample. Nonetheless, the population targeted for CES are all community members living in the five biometric woredas who are eligible for treatment. Thus, the CES denominator can be considered a representative sample of the Geshiyaro denominator and therefore coverage data similar enough to display concomitantly with Geshiyaro coverage.

## Conclusion

The main conclusion of this study is that aggregated data on MDA coverage and associated infection levels can give misleading information on which to base decisions on whether or not to stop prophylactic mass treatment to control STH infections. Finer spatial scale data reveals great heterogeneity, even when the overall mean coverage is high. The appropriate scale on which to report data and judge success, or failure, will vary by infection type and social setting. Both influence the scale on which transmission takes place and with what frequency. For many non-vector borne NTDS–the most important scale is likely to be within household and within village. However, the importance of between village and within district transmission is very poorly understood for most parasitic infections. Molecular epidemiological methods could help to reveal more about ‘who infects whom’ but to date these have not been employed in epidemiological research on the human helminth parasites.

## Supporting information

S1 Fig Comparison of MDA coverage estimates from the two data sources stratified by district and age group. (PDF)
