# Supplementary figures and images for: Spatial heterogeneity in mass drug administration from a longitudinal epidemiological study assessing transmission interruption of soil transmitted helminths in the Wolaita zone of southern Ethiopia (Geshiyaro Project)
Source: PLoS Negl Trop Dis. 2024 Feb 8;18(2):e0011947. doi: 10.1371/journal.pntd.0011947 (PMC10880954; doi:10.1371/journal.pntd.0011947)

**Difference in MDA coverage by data source**

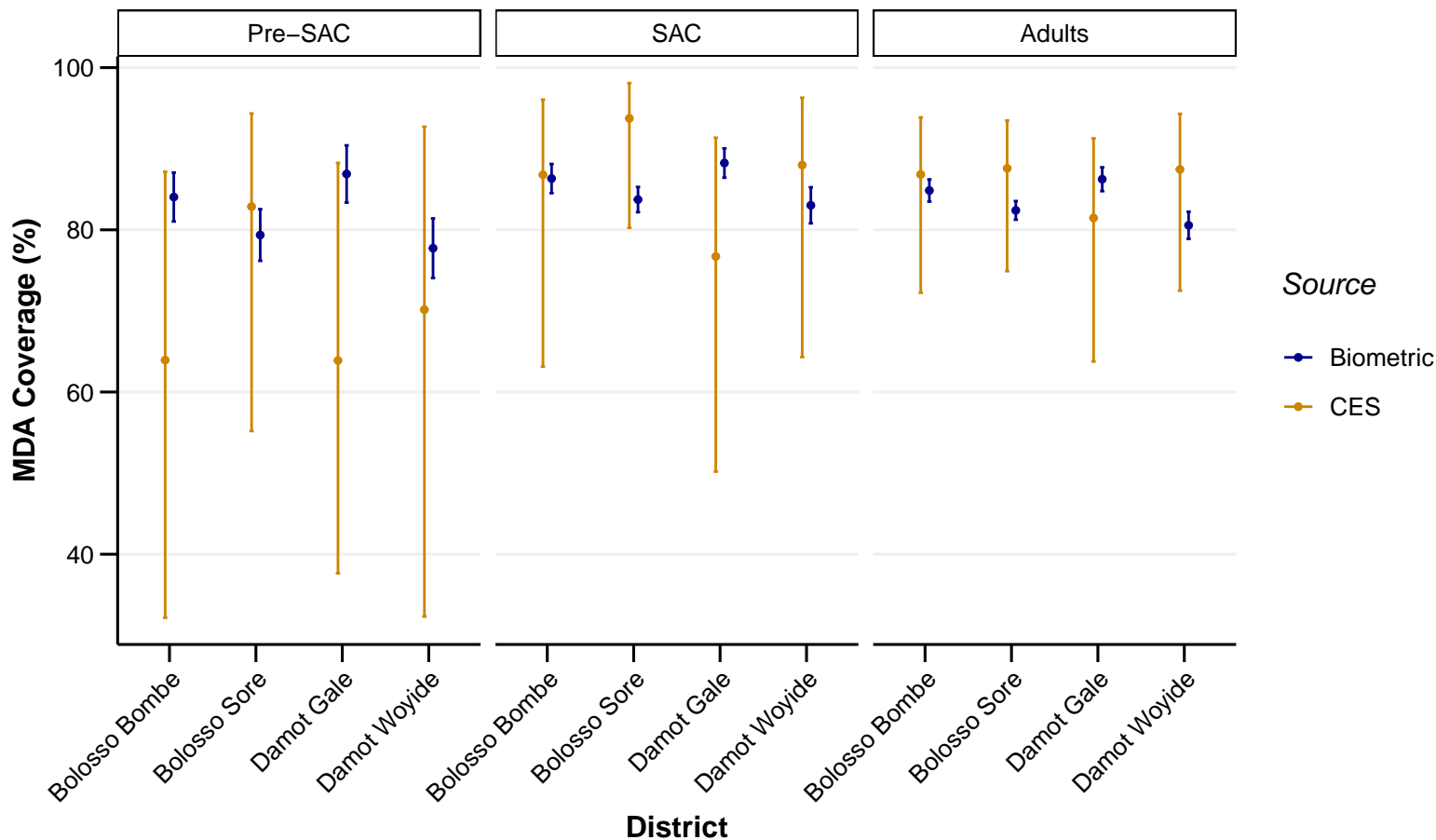

Supplement: S1 Fig — (PDF) [file pntd.0011947.s001.pdf]
